# Clinical Characteristics of Autism Spectrum Disorder in Israel: Impact of Ethnic and Social Diversities

**DOI:** 10.1155/2015/962093

**Published:** 2015-04-23

**Authors:** Muhammad Mahajnah, Rajech Sharkia, Haitham Shalabe, Ruth Terkel-Dawer, Ashraf Akawi, Nathanel Zelnik

**Affiliations:** ^1^The Child Neurology and Development Center, Hillel-Yaffe Medical Center, 38100 Hadera, Israel; ^2^The Ruth and Bruce Rappaport Faculty of Medicine, Technion, 33705 Haifa, Israel; ^3^The Triangle Regional Research and Development Center, P.O. Box-2167, 30075 Kfar Qari', Israel; ^4^Beit-Berl Academic College, 44905 Beit-Berl, Israel; ^5^The Child Neurology and Development Center, Clalit Health Services, Haifa District and Carmel Medical Center, 34362 Haifa, Israel

## Abstract

Despite the increased global prevalence and recognition of autistic spectrum disorder (ASD), it is still scarcely reported in the Arab world. Though Israel has a higher prevalence of ASD, a previous national survey of patients diagnosed between 1972 and 2004, demonstrated that 98% of them were of Jewish ancestry. The disproportional low number of Arab children with ASD in Israel is unclear but may reflect lower awareness and cultural bias. In the present study we collected clinical and demographic characteristics of 200 children with ASD from Arab and Jewish sectors in Israel that were evaluated in two child development centers. We compared the incidence and the medical comorbidity of autism between these two ethnics groups. The medical and psychiatric comorbidity profile in these children was similar to the worldwide published studies. In the present study the prevalence of autism in the Arab sector in Israel was similar to that of the Jewish sector. The Arab patients presented with more severe autistic manifestations and higher incidence of mental retardation, familial members with autism, and consanguinity (*P* < 0.05), while in the Jewish sector milder forms (such as Asperger syndrome and PDD-NOS) were more frequent. This discrepancy might be explained by both genetic and cultural factors.

## 1. Introduction

Autism is a medical term that encompasses a broad spectrum of neurodevelopmental disorders characterized by impaired reciprocal socialization and communication, often accompanied with stereotyped ritualistic behavior. Despite a strong genetic factor [1], there are no clear cut biological markers of autism and its diagnosis is purely based upon clinical observation, psychological tests and employment of diagnostic instruments, and standard checklist questionnaires, which are amenable to human bias [2, 3]. The clinical boundaries of autism spectrum disorder (ASD) have been changed over the years by the American Psychiatric Association and at present are based on the 5th edition of the Diagnosis and Statistical Manual of Mental Disorders (DSM V) [4]. The dramatic worldwide increase in the incidence of ASD during the last two decades might be related to environmental factors but is also a result of more permissive clinical criteria as well as increased worldwide awareness of ASD by both medical and educational personnel [5–7]. This awareness has expanded throughout the whole world, including the Middle Eastern Arab world, where autism was rarely reported in the past [8, 9]. In comparison to other Middle Eastern countries, Israel has a higher prevalence of ASD and, as in the rest of the Western world, the prevalence and incidence of ASD is higher than previously reported, though still lower than in USA [10]. A national survey that included Israeli patients diagnosed with ASD between the years 1972 and 2004 demonstrated that 98% of them were from Jewish ancestry [11]. The disproportional low number of children from Arab communities in Israel is unclear but may reflect lower awareness and cultural bias rather than genuine genetic diversity. Similarly, and probably from similar reasons, studies from USA have shown a lower prevalence of ASD is the Hispanic population in comparison to white Americans [12].

Studies comparing prevalence in different ethnic groups can provide a better understanding of neurobehavioral disorders such as autism and can further clarify the effect of various factors such as genetic and environmental factors on its pathogenesis. The Arab community comprises approximately twenty percent of the population in Israel and has specific social and environmental characteristics. During the recent decades there has been a change in the social structure of Arab society including alteration of some properties such as the decrease in the number of children in the family, improved economic status with more participation of women in the labor market, and increased awareness of neurobehavioral disorders. We assume that these changes in Arab society can explain the increasing morbidity and comorbidity of various neurobehavioral disorders including autism.

The present study aims to compare the clinical characteristics of children from Arab and Jewish communities in Israel and further assess the effect of genetic versus cultural and social factors.

## 2. Materials and Methods

Participants were 200 children with ASD diagnosed during the last five years (2008−2013) at two Child Neurology and Development centers in the Haifa and Hadera districts at the northwestern region of Israel (including Hillel-Yaffe Medical Center and Clalit Health Services in Haifa). These two centers cover a population of nearly 1,000,000 persons. All patients underwent a complete work-up and evaluation. Children with additional medical conditions that may contribute to neurologic dysfunction such as Down syndrome, fragile-X syndrome, significant premature birth (<34 weeks of gestation), birth asphyxia, cerebral palsy, Rett syndrome, and children with other chromosomal aberrations were excluded.

The diagnosis of ASD (autism, pervasive developmental disorders-not otherwise specified (PDD-NOS), or Asperger disorder) was based on fulfilling the required criteria of the* Diagnostic and Statistical Manual of Mental Disorders, Fourth Edition* (*DSM-IV*) and usage of additional diagnostic instruments, primarily the Childhood Autism Rating Scale (CARS) questionnaire [13]. Eligibility for diagnosis of ASD requires clinical evaluations by both a pediatric neurologist experienced in neurodevelopmental disorders and a developmental psychologist.

The clinical data was collected retrospectively from the medical records of these patients. Where necessary for completion of missing data, we contacted the parents and caregivers of the patients. The questionnaires included information about the medical and social background of the patients including ethnic identity, socioeconomic status, and educational and medical data retrieved from developmental rehabilitation institutions (as shown in Study Questionnaire).

In respect to the data that was summarized, developmental regression was defined as loss of any skill that was used consistently for at least 2 weeks and subsequently lost. These skills included all features of language and speech, eye contact, gestures, pointing, and fine motor skills.

In this study comorbid psychiatric disorders included mood disorders and aggressive/self-injurious behavior. Identification of mood disorders in individuals with autism spectrum disorders was based on clinical features of mood fluctuation and somatic signs of affect disorders according to* DSM IV* criteria. The symptoms of mood disorder must have been present intermittently or persistently for an estimated period of at least 6 months. Aggressive/self-injurious behaviors were defined as unprovoked kicking, biting, pinching, hitting, or head-banging toward self or others that occurred daily and were persistent for an estimate of 6 months or more.

The database was created in Access software (Microsoft Inc, Redmond, Washington). The data were subject to random spot-checking and verification and were imported into SPSS software (SPSS Inc, Chicago, Illinois) for statistical analysis. Frequency tallies were performed on all categorical variables, prevalence rates determined, and *χ* ^2^ tests carried out as appropriate.

## 3. Results

Approximately 4,300 pediatric patients are referred annually to both child development and pediatric neurology centers for assessment of neurodevelopmental difficulties. Out of them 69% are Jews, while the rest (31%) are Arabs. Among them, within the last five years (2008–2013) 200 children were diagnosed with ASD and were included in the current study. In this context, data retrieved from the official website of the Israeli Central Bureau of Statistics (ICBS) showed that during 2011-2012 the number of children aged 0–7 years in our study area was 120,822 (73,900 Jews, 46,922 Arabs) [14]. Taking into consideration that approximately 40% are referred to our two child development centers (the rest belong to different healthcare insurance corporations) means that in reality our centers provide medical service for 29,560 Jewish children and 18,768 Arab children. Subjects were grouped into two groups according to their ethnicity: a Jewish group (*n* = 129, 65%) and an Arab group (*n* = 71, 35%). Accordingly, the calculated estimated prevalence of ASD in our area was 4.3/1000 in the Jewish sector and 3.8/1000 in the Arab sector.

Table 1 contains selected characteristics of the 200 children with ASD and for the each group separately. The male to female ratio of the total sample was 7.3 : 1. Among Jews this ratio was higher (10.76 : 1) than among Arabs (4.4 : 1) (*P* = 0.037). The mean age of diagnosed patients was 37.2 ± 16.3 months (range 30.9 to 53.5) with no significant difference between Arabs and Jews. In both communities, most children were diagnosed with ASD before school entry, indicating an increased awareness of autistic disorders at early age. The data showed that there is a significant difference between Arabs and Jews regarding relatives with Autism (17% versus 8.5%) and consanguinity (21.1% versus 2.3%), respectively. The course of pregnancy and birth were also investigated and we found that while vaginal delivery was more common in Arab women, caesarian section and vacuum extraction were more commonly among Jewish women (Table 1).

The different subtypes of ASD according to the DSM IV classification among Jewish and Arab patients are shown in Table 2. While the prevalence of “classic” autistic disorder was relatively higher among Arab patients, both Asperger's disorder and PDD-NOS were relatively more prevalent in the Jewish patients. The majority of our patients were diagnosed as a primary autism (68.5%) with the others as secondary regression. Additionally, there was no significant difference between Jewish and Arab patients according to this type of diagnoses. In this study the mean CARS scale was quite high (35.38), with Arab patients getting higher score on the scale than Jewish patients, indicating more severe autistic manifestations in the former group.

The prevalence of associated disorders that are found in children with ASD in the present study is shown in Table 3. The most common comorbid disorder is sleep disturbance (40.5%) without difference between Arab and Jewish patients. Interestingly, among Jewish patients the second and third common concurrent disorders were found to be anxiety (36.4%) and behavioral difficulties (19.4%), whereas among Arab patients behavioral difficulties (32.4%) and anxiety (16.9%) were observed as the second and third comorbid disorders, respectively. Furthermore, mental retardation and epilepsy were found to be the fourth and the last concurrent disorders with autism among all patients with significant differences between Jew and Arab patients. Jewish autistic patients have more incidence of epilepsy (9.3% versus 4.2%) than the Arab autistic patients whereas Arab autistic patients have more incidence of mental retardation (14% versus 8.5%).

Table 4 shows the persons who first raised the possibility of the children having an autistic disorder and selected clinical presentation among our patients. It demonstrates that parents were the first to suspect that their children have autistic disorder (69%) in both Jewish and Arab communities. Among Jewish patients, kindergarten teachers more commonly suspected the autistic disorder before the patient reached a physician, whereas among Arab patients this was not the case. Interestingly, it was found that the first and the second common symptoms of ASD among Jewish patients were language delay (35.5%) and communication deficits (25%), whereas among Arab patients communication deficits (35.21%) and language delay (29.75%) were found to be the first and the second common symptoms of ASD, respectively. Additionally, a significant difference was found between both groups regarding communication deficits symptoms. Other clinical symptoms did not show any significant differences between the two groups.

*Study Questionnaire: Autistic Spectrum Disorder—Clinical Data*

*(A) General Information*

Date: …/…/…

- Subject ID: … Date of birth/Age: … Gender: M/F
- Birth history: Week of gestation: …, Birth weight: … (Grams), Mode of delivery: …
- Apgar score: 1′ … 5′ …
- Gestational complications: □ No □ Yes
- If yes, specify: …
- Delivery complications: □ No □ Yes
- If yes, specify: …
- Neonatal medical complications: □ No □ Yes
- If yes, specify: …

*(B) Diagnosis of Autism*

- Age of onset of symptoms: …
- Age of diagnosis: …
- The diagnosis was determined by:
  1. Professional physician
  2. Professional psychologist
  3. Other
- The diagnostic instruments:
  1. □ DSM IV
  2. □ CARS
  3. □ ADOS
  4. □ Other
- (IVa) DSMIV Score: …
- (IVb) CARS Score: …

*(C) Symptoms and Clinical Comorbidity of the Autistic Child*

- PDD subtype:
  1. □ Autism
  2. □ Asperger syndrome
  3. □ PDD-NOS
  4. □ Other
-
   
  1. Primary autism
  2. Secondary regression
  3. Unknown
- Mental retardation: □ No □ Yes
- If yes: (1) Mild, (2) Moderate, (3) Severe, (4) Unknown
- Sleep disorder/insomnia: □ No □ Yes
- If yes: (1) Difficulties in falling asleep, (2) Difficulties during sleep, (3) Excessive night arousals, (4) Difficulties in morning arousal, (5) Unknown
- Gastrointestinal symptoms: □ No □ Yes
- If yes: (1) Diarrhea, (2) Constipation, (3) Vomiting, (4) Food intolerance, (5) Abdominal pain, (6) Other
- Epilepsy or seizures: □ No □ Yes
- If yes:
  1. Age of onset: …
  2. Epilepsy type:
    1. Generalized
    2. Simple partial
    3. Complex partial
    4. Partial with secondary generalization
    5. Single episode
    6. Others: specify: …
- Behavioral and psychiatric disorders
  1. Anxiety: □ No □ Yes □ Unknown
  2. If yes please details: …
  3. Depression: □ No □ Yes □ Unknown
  4. If yes please details: …
  5. Obsessive compulsive disorder: □ No □ Yes □ Unknown
  6. If yes please specify: …
  7. ADHD: □ No □ Yes □ Unknown
  8. If yes please specify: …
  9. Other psychiatric disorder: □ No □ Yes □ Unknown
  10. If yes please specify: …
- Other chronic diseases: □ No □ Yes □ Unknown

*(D) Ethnic Group*

(II) Jewish Subgroup: …
(III) Arab Subgroup: …
(IIII) Other: …

*(E) Familial Status*

- Parental consanguinity: □ No □ Yes
- Consanguinity degree: …
- Adopted child
- Parental status: (1) Married, (2) Divorced, (3) Uniparental family
- Number of children in the family: …
- The serial number of the autistic child in the family: …
- Is there any neurologic, genetic, or developmental disorder/disease in the family? □ No □ Yes
- If yes, please specify: …

*(F) Socioeconomic Status*

- Father education: …
- Mother education: …
- Father employment status/occupation: …
- Mother employment status/occupation: …

*(G) The Educational Status of the Autistic Child*

- Private day-care center
- Private regular kindergarten
- Public regular kindergarten
- Special education kindergarten
- Regular school class
- Regular school class with assistance
- Special education school class
- Other: …
- None
- Unknown

Notes: …

## 4. Discussion

ASD is a complex neurodevelopmental disorder characterized by qualitative impairments in three domains: social interaction, communication, and repetitive, stereotyped behavior. As a rule, these impairments begin in early childhood (before the age of three years), persist throughout the full life span, and often cast a detrimental impact on the well-being of affected individuals [15]. Similarly to other developmental brain disorders it is commonly associated with other medical and psychiatric comorbidities including behavioral difficulties, sleep disorders, gastrointestinal disorders, and epilepsy.

In the recent years there has been a significant global increase in the incidence of ASD though most reports come from the Western world. This phenomenon is attributed to environmental and genetic factors [16–18]. More genes are being discovered and considered as contributing to the development of autistic disorders and recent studies indicate a form of complex genetic inheritance [19]. In addition, increased parental age, specially the paternal age, may also contribute to the increased incidence of ASD [20].

In contrast to the global increase in the awareness and prevalence of autism, publications in this field in the Arab world over a 25-year period were found to be underrepresented [21]. Relatively little is known about the clinical correlation and comorbidity of autism in Middle Eastern and Arab countries [22, 23]. In Saudi Arabia, one study investigated 49 patients with autism and found that females were older than males at the time of referral, 11 patients had a history of seizure disorder, 25 patients were taking psychotropic medications, and 14 patients were the product of consanguineous marriages [24]. In a study from the United Arab of Emirates (UAE), a representative random sample of 694 three-year-old children with ASD was evaluated in a two-stage study in the community. The weighted prevalence was estimated to be 29–58 per 10,000 for a DSM-IV diagnosis of pervasive developmental disorder (PDD). The presence of autistic features was associated with male gender, the presence of behavioral problems, and a family history of developmental delay [8]. A recent study of ASD in Arab Countries recruited a total number of 37 boys and 23 girls from three Arab countries (Egypt, Saudi Arabia, and Jordan) and found that the boys had poor emotional responsiveness and the girls had more cognitive problems [25]. In this study the mean age of diagnosis was relatively high (8.6 years in the Egyptian patients, 8.2 years in the Saudi patients, and 7.9 years in the Jordanian patients) the percentage of the female patients was relatively high (52% in the Egyptian patients, 21% in the Saudi patients, and 40% in the Jordanian patients). Another recent study from Lebanon investigated the association of autism with several factors in 86 children of autism from specialized schools for children with developmental disabilities and 172 control children from regular public school in the same regions [26]. They found that male sex, older age, living close to industrial compounds, previous childhood infection, and maternal depression during pregnancy were all associated with autism.

Our study is a unique epidemiological and clinical research in several ways. It is a comparative study that characterizes the clinical features and comorbidities of two different ethnic groups, Jews and Arabs, living in the same region with access to the same medical services. These ethnic groups have different social and cultural features with different genetic background. Comparing these two ethnic populations can underscore the effect of genetic versus cultural or environmental factors in the prevalence of ASD and its comorbidities. Our results show that the prevalence of the ASD among the Arab population in Israel is proportional to that within the Jewish population. While the estimated prevalence of ASD in our area (4.3/1000 and 3.8/1000 among Jewish and Arab, resp.) is still lower than in the Western world, it is in accordance with, though somewhat lower than, the prevalence that was recently found by Davidovitch et al. [10]. These results contradict a previous national survey that included Israeli patients diagnosed with ASD between the years 1972 and 2004 demonstrating that 98% were from Jewish ancestry and only 2% were Arab [11]. Hence, the present study shows a sharp increase of ASD diagnosis in the Arab population during the last decade. Several factors contributed to this increase including a raised social awareness in parents and school and kindergarten staff and increased accessibility to pediatricians, pediatric neurologists, and pediatric psychiatrists specializing in childhood neurodevelopmental disorders. These results also indicate that the social, environmental, and cultural factors interplay a critical role in the etiology of ASD. We assume that during the last decade fundamental social changes occurred in the Arab population including paternal age, women labor, socioeconomic status, and lifestyle. All of these factors can contribute to the increase of the ASD. It is implausible that genetic factors are responsible for this increase because it is unlikely that significant genetic alterations occurred during such a short time (10 years).

We also investigated the question of who was the first to suspect the diagnosis of ASD in the child. Jews and Arab parents alike were the first to raise this suspicion. For Jewish patients kindergarten staff suspected ASD while in Arabs patients this was not the case and only their physicians raised the issue. These results suggest that the awareness of ASD is higher among the teaching staff in the Jewish community than in the Arab community. More efforts are required in order to raise the awareness among the school and education staff within the Arab community.

*Limitation of the Study*. The main drawback of the study is its retrospective nature. Hence the data was collected from medical files and parents interviews. Although all patients reside in the Haifa and Hadera districts, not all of them were referred to our two Child Development centers. Furthermore, the children that are referred to the Child Development centers are mostly preschool children. Thus there is a possibility that a substantial number of children, who were diagnosed as autistic later in life (during school years), were not included. Though we made an effort to calculate the estimated prevalence of ASD in our regional area, this is still not a national population-based survey and therefore does not allow us to accurately estimate the prevalence of ASD in Israel in either the Jewish or the Arab sectors of the population. There is a need to conduct a more comprehensive national survey of all the patients with autism in Israel, particularly in older age, in order to study the role of genetic, environmental, and social effects on the diagnosis of ASD.

## 5. Conclusions

The present study demonstrates that while the prevalence of ASD is similar in both Arabs and Jews, Arabs were more commonly diagnosed with autistic disorder (including cases with severe autism who received a higher clinical CARS score). In contrast, milder subtypes of ASD (such as Asperger syndrome and PDD-NOS) were more commonly found in Jewish patients. Likewise, mental retardation was also more commonly found among the Arab patients. The reason for this discrepancy is not entirely clear but is conceivable that parental consanguinity and perhaps lower index of suspicion in milder cases contribute to this phenomenon. In this respect, despite the increase in the socioeconomic status and education of the Arab community in Israel consanguinity is still relatively common [27]. We believe that it also plays a major role in the fact that ASD was more frequently found in siblings and family relatives among the Arab patients.

## Figures and Tables

**Table 1:** Clinical characteristics of the 200 children with autism spectrum disorders. Significant differences (*P* value <0.05) were found between the two ethnic groups in patient gender, course of delivery, and consanguinity (see text for details).

Characteristic	Total number (%)	Jews number (%)	Arabs number (%)	*P* value
Male	176 (88)	118 (91.5)	8 (81.7)	0.037
Female	24 (12)	11 (8.5)	13 (18.5)	0.037
Age of diagnosis, mean months (±SD)	37.2 ± 16.3	38.5 ± 18.8	34.6 ± 12.11	0.11
First child	93 (46)	71 (55)	22 (30.5)	0.05
Relatives with autism	23 (11.5)	11 (8.5)	13 (17)	0.021
Consanguinity	18 (9.0)	3 (2.3)	15 (21.1)	0.0001
Vaginal delivery	114 (57.0)	67 (51.9)	47 (66.2)	0.004
Cesarean section	75 (37.5)	52 (40.3)	23 (32.4)	0.073
Vacuum delivery	10 (5.0)	9 (7.0)	1 (1.4)	0.020
Pregnancy complications	64 (32)	44 (34.10)	20 (28.16)	0.290

**Table 2:** The autistic subtypes of autism spectrum disorders among Jews and Arab patients. More Arab patients with autism-PDD than Jewish and more Jewish patients with Asperger and PDD-NOS than Arab (*P* < 0.05 statistical significance; see text for details).

	Total number (%)	Jews number (%)	Arabs number (%)	*P* values
(1) Type of autism disorder				
Autism-PDD	120 (60)	66 (51.2)	54 (76.1)	0.01
Asperger	9 (4.5)	8 (6.2)	1 (1.4)	0.0001
PDD-NOS	71 (35.5)	55 (42.5)	16 (22.5)	0.0001
(2) Clinical course				
Primary autism	137 (68.5)	93 (72.1)	44 (62)	0.11
Secondary regression	61 (30.5)	34 (26.4)	27 (38)	0.08
Unknown	2 (1)	2 (1.5)	0	0
(3) CARS scale	35.38 (±2.26)	33.8 (±5.7)	37.4 (±6.3)	0.001

**Table 3:** Prevalence of concurrent disorders in the study patients. More Jewish patients suffering from epilepsy and anxiety and more Arab patients suffering from mental retardation and behavioral difficulties (*P* < 0.05 statistical significance; see text for details).

Concurrent disorder	Total number (%)	Jews number (%)	Arabs number (%)	*P* values
Mental retardation	21 (10.5)	11 (8.5)	10 (14)	0.012
Sleep disturbance	81 (40.5)	50 (38.7)	31 (43.6)	0.299
Epilepsy	15 (7.5)	12 (9.3)	3 (4.2)	0.001
Anxiety	59 (29.5)	47 (36.4)	12 (16.9)	0.003
Behavioral difficulties	49 (24)	25 (19.4)	23 (32.4)	0.031

**Table 4:** Person who first suspected children to be having an autistic disorder and selected clinical presentation among study patients. Parents were the most common people who suspected autism among the two populations (*P* < 0.05 statistically significant; see text for details).

	Total number (%)	Jews number (%)	Arabs number (%)	*P* values
Parents	138 (69)	89 (68.9)	49 (69.1)	0.561
Kindergarten teacher	28 (14)	22 (17)	6 (8.5)	0.012
Doctor	31 (15.5)	16 (12.4)	15 (21)	0.06
Global delay	34 (17)	15 (11.26)	9 (12.67)	0.492
Language delay	71 (35.5)	50 (38.75)	21 (29.75)	0.12
Eye contact	28 (14)	20 (15.5)	8 (11.26)	0.27
Communication	50 (25)	25 (19.37)	25 (35.21)	0.01
Behavioral	9 (4.5)	5 (3.80)	4 (5.68)	0.4
Clumsiness	8 (4)	5 (3.87)	3 (4)	0.528
Regression	16 (8)	11 (8.52)	5 (7.04)	0.471
Convulsions	6 (3.0)	4 (3.0)	2 (2.8)	0.64
